# Effect of pH conditioners on tooth bleaching

**DOI:** 10.1002/cre2.172

**Published:** 2019-02-19

**Authors:** Yuki Ito, Masayuki Otsuki, Junji Tagami

**Affiliations:** ^1^ Cariology and Operative Dentistry, Department of Restorative Sciences, Graduate School of Medical and Dental Sciences Tokyo Medical and Dental University Tokyo Japan

**Keywords:** bovine tooth, hematoporphirin‐stained paper, pH conditioner, tooth bleaching

## Abstract

The purpose of this study was to evaluate the effect of pH conditioners on tooth bleaching using hematoporphirin‐stained paper and artificially discolored bovine tooth model. Experimental bleaching gels containing 23% hydrogen peroxide, adjusting pH 7.0 by different pH conditioners (NaOH, NaHCO_3_, Na_2_CO_3_, KOH, KHCO_3_, and K_2_CO_3_), were prepared. Each bleaching gel was applied on a hematoporphirin‐stained paper, and the light was exposed for 5 min. Before and after bleaching, color was measured and color difference was calculated. Artificially discolored bovine tooth samples were prepared and bleached by four experimental bleaching gels containing NaOH, NaHCO_3_, Na_2_CO_3_, or KHCO_3_. The bleaching time was 10 min with light exposure, and bleaching was repeated 10 times. The color of bleached surface was measured at each bleaching period, and color difference was calculated. In the experiment using hematoporphirin‐stained paper, degrees of color difference were KHCO_3_ > NaHCO_3_ > KOH > NaOH > Na_2_CO_3_ ≥ K_2_CO_3_. In the experiment using bovine teeth, degrees of color difference were KHCO_3_ > NaHCO_3_ > NaOH > Na_2_CO_3_. It was concluded that the bleaching materials with same pH and different pH conditioners showed different bleaching effects and that both cation and anion in the pH conditioners affected bleaching effect.

## 1 INTRODUCTION

Tooth bleaching is one of the most conservative and cost‐effective esthetic treatments, and it gives a patient beautiful smile with satisfaction (ADA Council on Scientific Affairs, 2009). The demand for tooth bleaching has been increased for several decades as increasing number of patients (ADA Council on Scientific Affairs, 2009; Kwon & Wertz, 2015). The high demand is also reflected in the distribution and use of various bleaching materials and products by dental professionals (Kwon & Wertz, 2015). There are two techniques of tooth bleaching methods as dental treatments. One is professionally applied in the dental office as in‐office bleaching, and another is dentist‐prescribed/dispensed and patient home‐use bleaching as at‐home bleaching (ADA Council on Scientific Affairs, 2009).

In‐office bleaching is applied in a dental office, and one or several visits are required to achieve satisfactory results. In‐office bleaching materials contain various concentration of hydrogen peroxide as an active ingredient. Although the basic mechanism of the bleaching process has not been well known, the mechanism of in‐office bleaching can be explained as reaction of hydrogen peroxide with chromogen, which causes discolored teeth (Claiborne et al., 2014; Lee et al., 2009). Molecules of hydrogen peroxide in the bleaching material are separated to water and oxygen molecules on the applied tooth surface. During this reaction, several kinds of free radicals, such as oxygen (O·), hydroxyl radical (OH·), perhydroxyl radical (HO_2_·), and super oxide anion (O_2_–·) were produced (Minoux & Serfaty, 2008), and they react with chromogen molecules on the surface and in the subsurface of the tooth substrate, and then those molecules are separated to the smaller transparent molecules.

The bleaching effect is affected by various factors such as the concentration of hydrogen peroxide, application period, and number of bleaching times (Buchalla & Attin, 2007). Also, the reaction of hydrogen peroxide is accelerated by higher temperature, catalyst, and higher pH. Higher temperature by irradiation of various lights showed higher bleaching effect, and the visible light activating titanium oxide photo catalyst with suitable wavelength of light was effective for tooth bleaching (Suyama et al., 2009). Higher pH of bleaching product showed also higher bleaching effect (Ito & Momoi, 2011).

Some bleaching products consist from two bottles or syringes. One contains hydrogen peroxide and another contains pH conditioner. At the tooth bleaching, two components are mixed, and mixed gel is applied on the tooth surface. Although the details of pH conditioner are not well disclosed by manufacturers, the pH conditioners of bleaching materials may be varied. The effects of various pH conditioners of in‐office bleaching materials on tooth bleaching are not well known. The purpose of this study was to evaluate the effect of pH conditioners on tooth bleaching using hematoporphirin‐stained paper and artificial discolored bovine tooth model.

## 2 MATERIALS AND METHODS

### 2.1 Experiment using hematoporphyrin‐stained paper

#### 2.1.1 Preparation of hematoporphyrin‐stained papers

The hematoporphirin‐stained paper was prepared according to previous studies as follows (Kusanagi et al., 2018; Suemori et al., 2008). The 0.24 g of hematoporphyrin powder (Wako Pure Chemical, Osaka, Japan) was dissolved in 300 ml of ethanol, and 0.1 wt% of hematoporphyrin solution was prepared. The photo printing paper (Canon, Tokyo, Japan) was immersed in the solution for 5 min and then naturally dried in a dark room. The stained paper was trimmed suitable size and covered with a masking tape with a 5 mm of diameter hole to fit the probe of a colorimeter. This procedure ensured measuring the same area before and after bleaching.

#### 2.1.2 Color measurement

The CIE L*a*b* values of surface of hematoporphirin‐stained paper were recorded prior to bleaching as a baseline using a colorimeter (NR‐11A, Nippon Denshoku, Tokyo, Japan), and then the photograph of each experimental surface was taken by a digital camera. In order to decrease the variation among the specimens, only the specimens which showed L* value between 48 and 52 were selected for the experiment.

#### 2.1.3 Preparation of bleaching material

The 10 ml of 35% hydrogen peroxide (Wako Pure Chemical), 5 ml of deionized water, and 0.35 g of carboxymethyl cellulose sodium salt (Wako Pure Chemical) were mixed. The carboxymethyl cellulose sodium salt was used as thickener to increase the viscosity of bleaching gel. Then each of sodium hydroxide (NaOH, Wako Pure Chemical), sodium bicarbonate (NaHCO_3_, Wako Pure Chemical), sodium carbonate (Na_2_CO_3_, Wako Pure Chemical), potassium hydroxide (KOH, Wako Pure Chemical), potassium hydrogen carbonate (KHCO_3_, Wako Pure Chemical), or potassium carbonate (K_2_CO_3_,Wako Pure Chemical) was added as a pH conditioner, and pH of the solution was adjusted as 7.0 (Table 1). Final concentration of hydrogen peroxide in the bleaching materials was 23.0%.

**Table 1:** Ingredients and concentration of pH conditioner (mmol/L) in each experimental group

Group	pH conditioner	Other ingredients	Concentration of pH conditioner (mmol/L)	pH	Experiment	
					HP	BT
NaHCO_3_	Sodium bicarbonate 0.33 g	35% Hydrogen peroxide 10‐ml Deionized water 5‐ml Carboxymethyl cellulose sodium salt 0.35 g (all groups)	260	7.0 (all groups)	○	○
NaOH	Sodium hydroxide 0.005 g		8.30		○	○
Na_2_CO_3_	Potassium sodium carbonate 0.01 g		6.20		○	○
KHCO_3_	Potassium hydrogen carbonate 0.30 g		200		○	○
KOH	Potassium hydroxide 0.01 g		10.0		○	x
K_2_CO_3_	Potassium carbonate 0.01 g		4.80		○	x

*Note*. BT: Experiment using artificially discolored bovine tooth model; HP: Experiment using hematoporphirin‐stained paper.

#### 2.1.4 Bleaching and color measurement

Each bleaching agent was applied on the surface of hematoporphirin‐stained paper and photo‐irradiated for 5 min using an LED light unit (Cosmo Blue, GC, Tokyo, Japan). The peak wavelength of the light unit was 405 nm, and its intensity was 55 mW/cm^2^. The irradiation time was determined by the result of a pilot study. After light exposure, bleaching agent was removed and color measurement and photograph were repeated. The number of specimen was 12 in each group (*n* = 12).

The difference of L*, a*, and b* before and after bleaching was expressed as ΔL, Δa, and Δb respectively. The color difference (ΔE) before and after bleaching was calculated according to the following equation:

$$\mathrm{\Delta E}={\left[{\left(\mathrm{\Delta L}\right)}^{2}+{\left(\mathrm{\Delta a}\right)}^{2}+{\left(\mathrm{\Delta b}\right)}^{2}\right]}^{1/2}.$$

#### 2.1.5 Statistical analysis

The ΔL, Δa, Δb, and ΔE values were statistically analyzed by two‐way analysis of variance (ANOVA) with factors of cations (sodium and potassium) and anions (hydroxide, carbonate, and bicarbonate) of pH conditioners. And they were further analyzed by one‐way ANOVA and Tukey's HSD test at confidential level of 95% (*P* = 0.05).

### 2.2 Experiment using bovine teeth

#### 2.2.1 Preparation of stained bovine teeth

**Figure 1:**
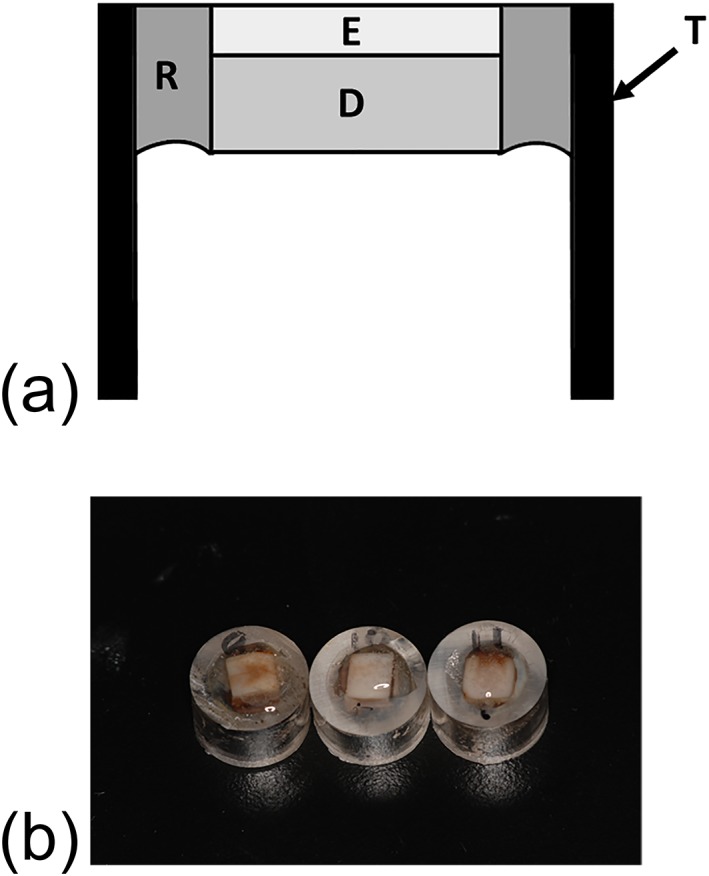
Specimens of artificially discolored bovine teeth. (a) Schematic illustration of the specimen. T: acrylic tube; R: quick self‐curing acrylic resin; E: enamel; D: dentin. (b) Photograph of the specimens

Artificially discolored bovine tooth samples were prepared according to previous studies as following method (Kusanagi et al., 2018; Kyaw et al., 2018). The crowns of extracted bovine incisors were cleaned by removing soft tissue remnants using a scalpel, and the labial surfaces were ground by #600 and then #800 silicon carbide (SiC) papers (Sankyorikagaku, Saitama, Japan) to obtain flat surfaces. Enamel thickness was kept approximately 1 mm. Then 6 mm × 6 mm size of specimens were obtained by cutting using a diamond cutter (Mini cutter machine MC−110, Maruto Instrument, Tokyo, Japan). Each specimen was set into a cylindrical acrylic tube with 10 mm of height and 10 mm of internal diameter and was fixed by a quick self‐curing acrylic resin (Unifast III clear shade, GC, Tokyo, Japan). At the setting, enamel surface was exposed to outside, and pulpal surface of dentin was exposed in the tube (Figure 1a). After curing, surface of specimens was further polished by #1,000 and #1,200 SiC papers. Then 5% sodium hypochlorite (Wako Pure Chemical) was applied on the pulpal side of dentin for 1 min to remove the organic remnants, followed by washing with running water. Then this surface was treated with 40% phosphoric acid gel (K‐etchant GEL, Kuraray Noritake Dental, Tokyo, Japan) for 10 s to open the dentinal tubules, followed by washing with running water. Finally, specimens were ultrasonically cleaned for 3 min.

#### 2.2.2 Staining the sample

Two tea bags (Lipton Yellow label tea bag, Unilever Japan, Tokyo, Japan) were immersed in 100 ml of boiled water for 10 min. The tea extract was used as staining solution. Specimens were immersed in the staining solution for 14 days at 37°C. The solution was changed at the fourth day. After staining (Figure 1b), the color of the surface of the specimen was measured by a colorimeter to obtained L*, a*, and b* values; the specimens which L* value showed between 50 and 60 were employed for the experiment; and the photograph of surface of each samples was taken by a digital camera.

#### 2.2.3 Tooth bleaching and color measurement

Based on the results of the experiment using hematoporphyrin‐stained paper, the bleaching agents containing NaOH, NaHCO_3_, Na_2_CO_3_, and KHCO_3_ were selected from six bleaching materials used for the experiment with hematoporphirin‐stained paper (Table 1). Effects of OH, HCO_3_, and CO_3_ can be compared among NaOH, NaHCO_3_, and Na_2_CO_3_ groups, and effects of Na and K can be compared between NaHCO_3_ and KHCO_3_, respectively. The method of preparation of the bleaching material was same as that of the experiment using hematoporphirin‐stained paper. The bleaching agent was applied on the surface of the specimen, and light was irradiated for 10 min using a same light unit as the experiment using hematoporphirin‐stained paper. After light exposure, bleaching agent was removed, the color was measured, and the photograph was taken. The bleaching and color measurement were repeated 10 times. The number of specimens of each experimental group was 10 (*n* = 10). The color difference before and after each bleaching time was calculated from obtained L*, a*, and b* values as well as hematoporphirin‐stained paper experiment.

#### 2.2.4 Statistical analysis

The ΔE values were statistically analyzed by two‐way ANOVA with factors of pH conditioners and bleaching times, followed by one‐way ANOVA and Tukey's HSD test. The confidential level of these statistical analysis was 95% (*P* = 0.05).

## 3 RESULTS

### 3.1 Experiment using hematoporphyrin‐stained paper

**Figure 2:**
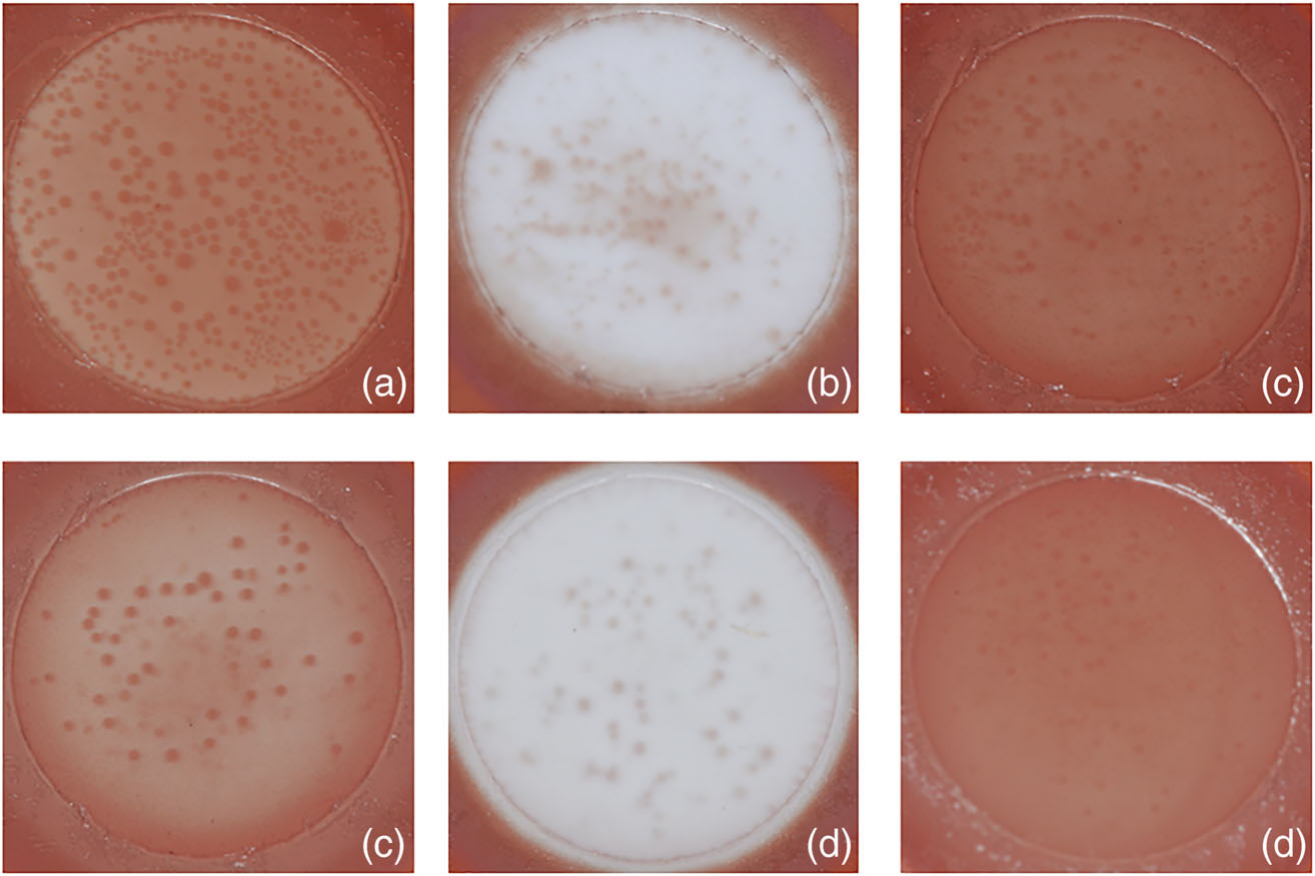
Typical images of hematoporphyrin‐stained papers after bleaching. (a) NaOH, (b) NaHCO_3_, (c) Na_2_CO_3_, (d) KOH, (e) KHCO_3_, and (f) K_2_CO_3_

Typical images of bleached samples in each group were shown in Figure 2. In all groups, samples showed bleaching effect. The mean values and standard deviations of ΔL, Δa, Δb, and ΔE in each experimental group were shown in Table 2. L* value was increased, and a* and b* values were decreased by bleaching in all groups. Both cations and anions were affected for ΔL, Δa, Δb, and ΔE, respectively (*P* < 0.05), and there was no interaction between cations and anions. The groups containing potassium showed higher bleaching effect than those containing sodium, and bicarbonate‐containing groups showed the highest bleaching effect followed by hydroxide‐ and carbonate‐containing groups (*P* < 0.05). Degree of change of ΔE was KHCO_3_ > NaHCO_3_ > KOH > NaOH > Na_2_CO_3_ ≥ K_2_CO_3_. There were statistical differences among all groups (*P* < 0.05) except between K_2_CO_3_ and Na_2_CO_3_ (*P* > 0.05).

**Table 2:** ΔL, Δa, Δb, and ΔE values of hematoporphyrin‐stained paper experiment

Group	ΔL	Δa	Δb	ΔE
KHCO_3_	37.8 (1.6)^c^	−30.1 (0.6)^a^	−26.3 (0.9)^a^	55.0 (1.6)^e^
NaHCO_3_	34.7 (1.3)^c^	−29.8 (0.9)^a^	−29.8 (0.9)^a^	52.2 (1.5)^d^
KOH	17.9 (2.1)^b^	−21.6 (1.2)^b^	−17.5 (0.8)^b^	33.5 (2.0)^c^
NaOH	8.0 (1.4)^a^	−18.9 (0.6)^c^	−25.1 (1.8)^b^	27.4 (1.5)^b^
Na_2_CO_3_	6.1 (6.4)^a^	−17.3 (1.9)^d^	−15.0 (1.8)^c^	24.5 (1.7)^a^
K_2_CO_3_	5.5 (1.9)^a^	−18.5 (1.9)^d^	−15.3 (3.2)^c^	23.0 (1.7)^a^

*Note*. Same superscripts in each column show no significant difference.

### 3.2 Experiment using bovine teeth

**Figure 3:**
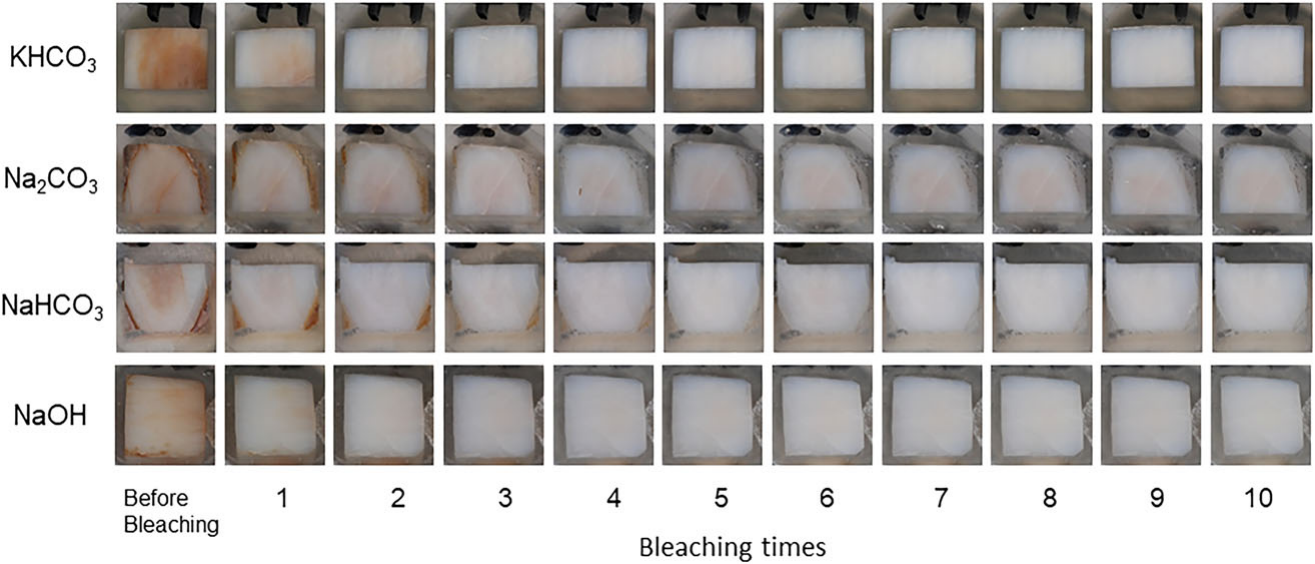
Typical images of bleached bovine samples at each period

**Figure 4:**
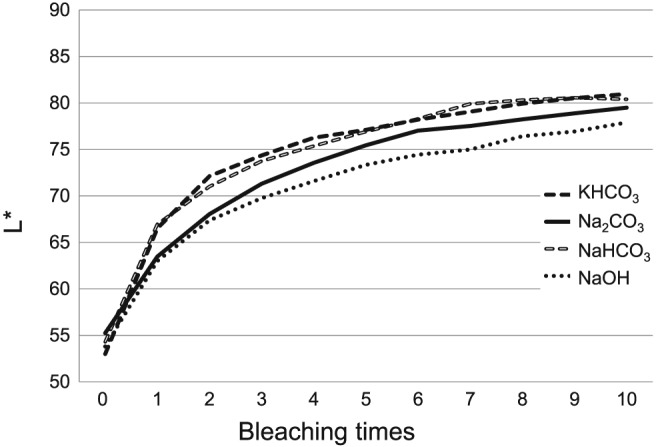
Change of L*a*b* and ΔE values of bleached bovine samples at each period. (a) Change of L* values, (b) change of a* values, (c) change of b* values, and (d) change of ΔE values

Typical images of the samples at each bleaching step in each group were shown in Figure 3. The specimen in all groups showed bleaching effect. Changes of L*, a*, b*, and ΔE in each experimental group were demonstrated in Figure 4. L* value was gradually increased, and a* and b* values were decreased in all groups by repeating bleaching procedure. Both factors (pH conditioners and bleaching times) were affected for ΔE (*P* < 0.05), and there was no interaction between them. Degree of change of ΔE was KHCO_3_ > NaHCO_3_ ≥ NaOH ≥ Na_2_CO_3_, showing statistical differences (*P* < 0.05) except between NaHCO_3_ and NaOH groups and between NaOH and Na_2_CO_3_ groups (*P* > 0.05).

## 4 DISCUSSION

There are many methods for evaluating bleaching effect. In this study, hematoporphirin‐stained paper and artificially discolored bovine teeth were used for evaluation of bleaching effect. Both experimental methods were previously employed for the research on tooth bleaching (Kishi et al., 2011; Kusanagi et al., 2018; Suemori et al., 2008). The evaluation using hematoporphirin‐stained paper is sensitive and seems to be suitable for a screening test. However, it is difficult to predict the bleaching effect from only the results of evaluation by hematoporphirin‐stained paper. It is necessary to evaluate the bleaching effect using teeth. Extracted bovine incisors were employed in this study as substitute of human teeth, because it is very difficult to collect enough numbers of extracted human incisors, and those were varied in the degree of discoloration that might affect the results of the experiment. It is easier to collect bovine incisors than human teeth with good condition, and they were already used for many *in vitro* tooth bleaching studies (Kishi et al., 2011; Kusanagi et al., 2018; Kyaw et al., 2018). Tooth discoloration is classified as extrinsic and intrinsic (Hattab et al., 1999). Because original extracted bovine teeth were very bright and whitish, they were stained by black tea extract for 14 days before the experiment from the surface and pulp chamber simulating extrinsic and intrinsic discoloration. Tea extract was used as the staining medium in previous studies (Kishi et al., 2011; Kusanagi et al., 2018; Kyaw et al., 2018; Sulieman et al., 2003), as tea is one of the typical extrinsic chromogens and is easily available. Stains produced by tea also have no potential for calcification, and the stains produced are easy to standardize, reproduce, and control (Sharif et al., 2000).

The accurate measurement of the tooth color is important for the evaluation of the tooth bleaching effect. Two kinds of methods are available for tooth color measurement. One is visual comparison between the target tooth and the tooth shade guides. Another is the measurement using a color measuring devise, such as a colorimeter or a spectrophotometer. In this study, a colorimeter (NR‐11) was used for color measurement of both hematoporphirin‐stained paper and a stained bovine tooth sample, as the color measurement by a colorimeter is able to obtain the objective results.

The pH of some bleaching products ranged from 3.7 which was highly acidic to 11.1 which was highly basic (Price et al., 2000). Among them, in‐office bleaching products had a pH between 3.67 and 7.85 (Majeed et al., 2011; Price et al., 2000). The bleaching material of higher pH showed greater bleaching effect with lower erosion than that of lower pH (Ito & Momoi, 2011). The active ingredient of the most in‐office bleaching products is hydrogen peroxide. Generally, the bleaching materials should storage in low pH condition and should be used in high pH to acquire the high bleaching effect. During the reaction of hydrogen peroxide in the bleaching material, the different pH produced different amount and kind of free radicals (Sharma & Sharma, 2017). Those difference caused the different bleaching effects (Ito & Momoi, 2011). In this study, all bleaching gels were adjusted as same pH (pH 7.0) to eliminate the effect of different pH on bleaching results.

Hydrogen peroxide solutions are more stable at lower pH. Most commercially available hydrogen peroxide solutions contain stabilizers (chelating and sequestering agents) to reduce decomposition of the product through transport and storage. The most stabilizers, such as phosphonic acids, are acidic and exhibit buffering properties, which add acidity to the product. Although the manufacturers do not disclose in detail about stabilizers, the amount and type of stabilizers may vary among products. Recently, some bleaching materials consist of two syringes. One syringe contains various concentration of hydrogen peroxide, and another contains pH conditioner (or accelerator), thickener, and additional catalyst and/or dye. The bleaching gel component that contains hydrogen peroxide has an acidic pH in most cases to be stable for storage reducing peroxide decomposition in an acidic environment. It appeared that the main function of the activating gel component synonymously referred to as catalyst or booster is to increase the pH of the mixed gel, thereby increasing the decomposition rate of peroxide and the formation of bleaching active radicals (Buchalla & Attin, 2007). Previous *in vitro* studies reported the effect of low‐pH bleaching material on the applied enamel surfaces (Soares et al., 2016; Sun et al., 2011; Xu et al., 2011). Theoretically, acidic bleaching materials have possibility to decalcify the enamel surface. Demineralized enamel would reduce mechanical properties and increase surface roughness. From this point, the bleaching material should not be acidic.

Many bleaching materials contain pH conditioner expecting increase of bleaching effect. In this study, the bleaching materials at pH 7 with different pH conditioner showed different bleaching effect. The pH conditioners used in this study were basic substances, which were separated to cations (Na^+^ and K^+^) and anions (HCO_3_^−^, OH^−^, and CO_3_^2−^) respectively in the hydrogen peroxide solution. Both cation and anion in the pH conditioner were affected bleaching effect. In the cations, the potassium ion (K^+^) was more effective than the sodium ion (Na^+^). In the anions, the bicarbonate ion (HCO_3_^−^) showed most bleaching effect followed by hydroxide ion (OH^−^) and carbonate ion (CO_3_^2−^). Although the mechanism of those difference was not clear, degree of ionization and ionization tendency of each ion might affect the reaction of hydrogen peroxide and showed different bleaching efficacy. The concentration of NaHCO_3_ and KHCO_3_ was 260 and 200 mmol/L, respectively (Table 1). Those concentrations were much higher than those of other pH conditioners. These difference might affect the bleaching effect.

Safety issue is clinically important. KOH and NaOH used as pH conditioners in this study are harmful and toxic as solid or solution of high concentration. However, their concentrations in the prepared bleaching materials are low, and pH is 7.0. It is thought that those bleaching materials evaluated in this study were not more toxic than the commercially available bleaching products with same concentration of hydrogen peroxide. The biocompatibility tests and clinical tests have to be required before clinical use of these materials. And the pH of the bleaching agents should be always checked, and if found to be acidic, the pH should be made neutral or alkaline, which may help prevent acidic damage to enamel and then to pulp by altering the bleaching chemistry (Sharma & Sharma, 2017).

## 5 CONCLUSION

Within the limitation of the study, it could be concluded that the bleaching materials with same pH and different pH conditioner showed different bleaching effect and that both cation and anion in the pH conditioner affected bleaching effect.

## CLINICAL SIGNIFICANCE

Not only pH of bleaching material but also selection of pH conditioner affects the tooth bleaching efficacy.
